# Applications of blockchain technology in peer-to-peer energy markets and green hydrogen supply chains: a topical review

**DOI:** 10.1038/s41598-024-72642-2

**Published:** 2024-09-20

**Authors:** G. B. Bhavana, R. Anand, J. Ramprabhakar, V. P. Meena, Vinay Kumar Jadoun, Francesco Benedetto

**Affiliations:** 1 Department of Electrical and Electronics Engineering, Amrita School of Engineering, Amrita Vishwa Vidyapeetham, Bengaluru, India; 2 Department of Electrical and Electronics Engineering, Manipal Institute of Technology, Manipal Academy of Higher Education, Manipal, Karnataka India; 3 SP4TE - Signal Processing for Telecommunications and Economics Laboratory, Economics Department, University of ROMA TRE, Via Silvio D’Amico 77, 00145 Rome, Italy; 4 Department of Electrical Engineering, National Institute of Technology Jamshedpur, Jharkhand, 831014 India

**Keywords:** Blockchain, Renewable energy, Microgrid, Smart grid, Green hydrogen supply-chain, Smart contracts, Renewable energy certificates, P2P trading, Engineering, Electrical and electronic engineering

## Abstract

Countries all over the world are shifting from conventional and fossil fuel-based energy systems to more sustainable energy systems (renewable energy-based systems). To effectively integrate renewable sources of energy, multi-directional power flow and control are required, and to facilitate this multi-directional power flow, peer-to-peer (P2P) trading is employed. For a safe, secure, and reliable P2P trading system, a secure communication gateway and a cryptographically secure data storage mechanism are required. This paper explores the uses of blockchain (BC) in renewable energy (RE) integration into the grid. We shed light on four primary areas: P2P energy trading, the green hydrogen supply chain, demand response (DR) programmes, and the tracking of RE certificates (RECs). In addition, we investigate how BC can address the existing challenges in these domains and overcome these hurdles to realise a decentralised energy ecosystem. The main purpose of this paper is to provide an understanding of how BC technology can act as a catalyst for a multi-directional energy flow, ultimately revolutionising the way energy is generated, managed, and consumed.

## Introduction

**Fig. 1:**
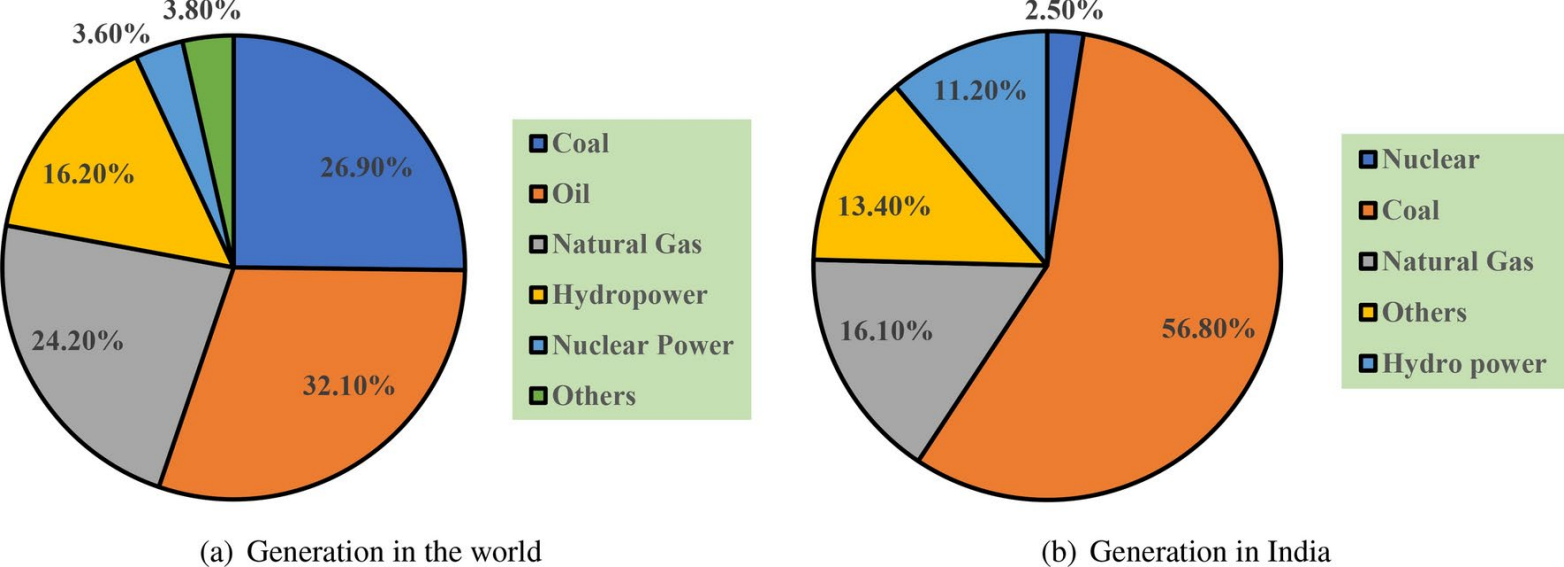
Major sources of energy.

As of 2023, the total electricity demand in the world was estimated at 28,000 TWh per year. This demand for power is growing steadily and is expected to be doubled by 2050. The demand rate differs from country to country and region to region^1^. Some have very rapid growth, and some might have steady growth. It depends on the rate of population increase, industrialization, and electrification. In India, the demand was 185 GW and is expected to go up to above 330 GW by 2030. This energy demand is met by conventional power systems that mainly employ non-renewable sources of energy. Figure 1a shows the share of primary sources of energy in the world, where oil is 32%, coal 26.9%, natural gas 24.2%, and the other energy sources are at 23.6%. Figure 1b illustrates the same but for India. As we can see from these two figures, the primary energy sources varies according to the geographical area of interest. The conventional energy system uses non-renewable sources of energy. Once used, they cannot be replenished within human lifetime, they cause pollution after combustion and hazards while extraction^2^. Their prices are volatile and are subject to political and geopolitical tensions and supply chain disruptions.

**Fig. 2:**
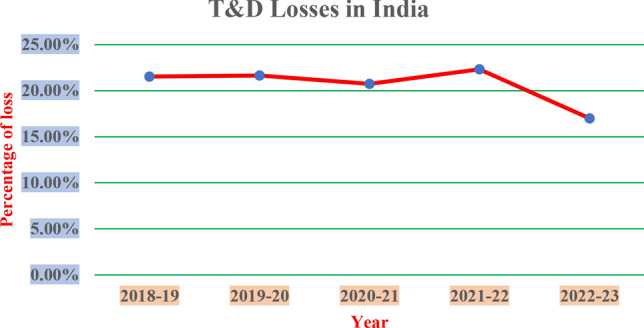
The transmission and distribution losses in India from 2018 to 23.

The conventional energy system also uses long-distance transmission lines for power distribution. These lead to energy losses during transmission^3^. The percentage of the losses varies from region to region. In India, according to the Central Electricity Authority, transmission losses were around 17% in 2023, see Fig. 2. The cost of construction and maintenance of long-distance transmission lines is huge (potentially millions and billions of dollars, depending on the distance). These transmission lines are prone to faults as a result of both environmental and non-environmental factors. These faults often cause disruptions in the electricity supply, causing inconvenience to consumers until the problems are rectified. The traditional system is also susceptible to the forking and stealing of electricity from the transmission lines. There is no way to verify the electricity the end user receives in these types of systems. The use and combustion of these fossil fuels result in pollution. This pollution is prompting the entire world to consider other energy sources.

Governments all over the world are pushing for greener and renewable sources of energy. In India, the government has started various initiatives like the national solar mission, national wind energy mission, green energy corridors, etc., to add 450 GW of RE production by 2030, increase renewable sources of energy, and achieve a target of producing 40% of its energy demand from them. RE sources are inexhaustible and do not result in any ecological harm or pose health risks. Their prices are relatively stable in comparison to their peers. Renewable energy is generated closer to the point of usage, and this eliminates the long-distance transmission lines and associated techno-economic challenges^4,5^.Renewable sources of energy are weather-dependent and volatile. Their fluctuations pose a threat to grid stability and may require added energy back up to have a consistent supply. They require specialised equipment for installation and technical ability for maintenance, making their installation costly and difficult for widespread adoption.

To effectively use RE and overcome volatility, the network participants should be able to use the electricity when it is produced or store it for future usage. Since effective energy storage mechanisms are not in place, the prosumers should be able to use that energy either by themselves or through a trade between the various entities involved in the microgrid. This trade can only be facilitated by a multi-directional flow and control of energy because, if the traditional transmission system is used, there can be various congestion points and the transfer of energy might take a very long time. Peer-to-peer trading can be employed to facilitate this trading effectively^6–8^. Through P2P trading, the prosumers (participants in the network who both create and use electricity) and consumers (participants who solely consume energy) engage in direct energy exchange with each other, bypassing the need to send it back to the grid. Local energy production and consumption minimise energy losses during distribution, hence enhancing system efficiency. It fosters market competition by offering consumers a wider range of options, enabling them to select a producer with more affordable energy rates. As a result, prosumers and producers lower the energy cost, enticing the consumer to select their services and, consequently, lowering energy costs. This would also enhance the likelihood of consumers transitioning into prosumers and generating their own electricity to fulfil their energy needs^9–12^.

A safe and reliable communication framework is necessary for this P2P trading. All network participants, particularly the consumers, ought to have access to real-time data on the electricity that all producers and prosumers in the network are producing or storing, as well as on the consumption patterns of the consumers. There may be some malicious network participants posing as producers to scam consumers for money. In order to minimise monetary losses, it is essential for the transfer of funds and electricity to occur simultaneously. P2P trading requires installation of smart meters and associated communication equipment, which makes installation costly. And it is difficult to manage the multi-directional flow of energy during trading. A proper regulatory, reliable, and secure communication platform is required to control the multi-directional flow of energy and avoid congestion in the lines. BC is an emerging technology that aims to ensure secure and transparent transactions because of its distributed and decentralised ledger. Data related to the chain and associated transactions of each block are stored on all the participating nodes, thereby eliminating the need for intermediaries^13–17^. These intermediaries in the traditional system lack accountability. Furthermore, they impose exorbitant fees for basic transactions. The presence of an intermediary leads to a longer duration of the transaction and amplifies the danger of security breaches. BC enhances the security and dependability of the system by eliminating these intermediaries. The integrity of the transaction records is inviolable, and their transparency mitigates the potential for corruption or fraud, fostering confidence among the network’s nodes^18–20^.

This article presents the four key application areas of BC in the energy sector, mainly: P2P energy trading, the green hydrogen supply chain, REC’s, and real-time DR mechanisms. In P2P trading, this paper compares the existing projects in that domain and identifies the merits and challenges. This paper also compares the various projects on BC being used in administrating the green hydrogen supply chain and explains the scenarios in which BC can be used and how it can maintain the security and traceability of the supply chain. BC is used to issue, verify and store the REC’s, as well as to trace and control the DR in the grid. It can also be used to incentivize consumers to participate in DR initiatives.

The paper is organised as follows: Section “Blockchain” explains the BC technology and its components, including SC as well as consensus protocols. Section “Microgrid” further elaborates on the benefits of microgrids in comparison with conventional grid systems and also includes the challenges and hindrances to the large-scale adaptation of microgrids. Proceeding further, section “Smart grid” describes smart grids and presents a comparison between smart grid, microgrid, and conventional grid. The various applications of BC pertaining to the energy sector are presented in section “Applications of blockchain in the energy sector”. In section “Conclusion”, we concluded the paper, identifying the key aspects, challenges, and future applications of this technology. Finally, in section “Future scope”, the future applications of BC in the energy markets and the required technological advancements are explained.

## Blockchain

**Fig. 3:**
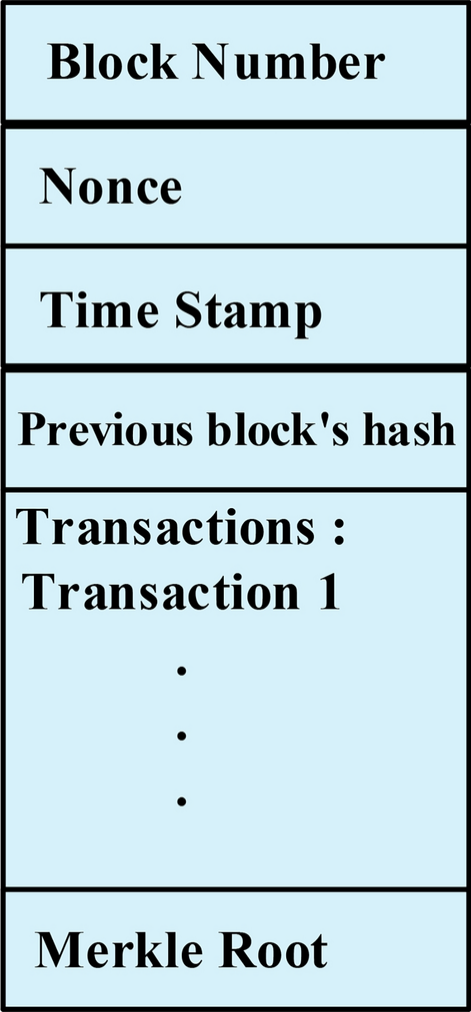
The components in a single block of a BC.

A cryptographically secure, distributed, immutable, and decentralised ledger that can only be appended is known as a BC. It operates as a P2P connection where updates are contingent upon consensus or agreement among the nodes. There is no central server or controller in a P2P network; therefore, all network participants can communicate directly with one another. A distributed ledger is maintained across all network participants. The entire ledger is replicated in its entirety across every node in the network. Utilising cryptography, a BC safeguards data from tampering and exploitation^21–23^. Input to the BC is restricted to a sequential, time-based progression. Once data is appended to a BC, it is almost impossible to modify that data unnoticed. Any modifications to the data require the approval of every node in the entire network. According to layperson terms, a BC is basically a chain of blocks or nodes linked together with a unique identifier value. Figure 3 elucidates the contents of a single block. A BC is made up of many such blocks.

### Blocks

- A block is basically a collection of transactions and is used to store data. Each block comprises of a body and a header. The header is made up of the time stamp, block number, merkel tree, nonce, and the preceding block’s hash. The block body includes all the transactions in that block. The leading block of the chain is designated as the genesis block, and it has NULL in place of the preceding block’s hash value.
- Nonce (number used only once) is a random number that helps find an exact hash that meets the pre-defined chain criteria. It is generated for every block during block creation. Miners (nodes that add new blocks to the chain) include the data of the new block along with a nonce to generate a hash. Each blockchain network has a specific difficulty level to mine new blocks, and only the hashes that meet this requirement are allowed to be included in the chain. Miners keep trying with a new nonce until the winning hash is obtained.
- A timestamp is used to establish the chronological order in which the transactions occur. It consists of the time and date at which the transactions are appended to the network. It helps protect the BC from repeating transactions and prevents tampering with the order of the transactions.
- Merkel tree, also called a hash tree, is the hash of all the hash values in that block. It contains the hashes of all the transactions and data within that particular block. Just by verifying the merkel tree of a block, all the data and the transactions in that block can be verified.

### Hash

BC makes use of hashes in order to establish privacy and security on the network. A hash is a unique digital fingerprint that is unidirectional and unique for all data. Even a minute change in the data can change the hash of that block entirely. The hash of a block is computed using all the data present in that block, including the entire transaction data of the block and previous block’s hash. The current block is linked to the previous block through the previous block’s hash stored in it. Once data is changed in the previous block, it’s hash changes, but it is different from the hash that is stored in the next block^24–26^. This mismatch breaks the link and notifies the network of a data breach, making it difficult to manipulate the data. Hashes are generated by cryptographic hash functions that take an input of any length and any data type and deliver an alphanumeric output of a fixed length.

### Consensus protocols

**Fig. 4:**
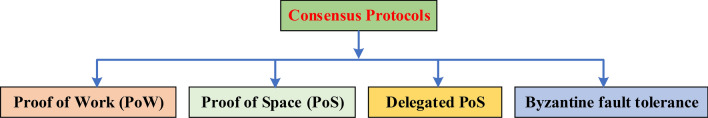
Consensus protocols used in BC.

Consensus protocols are used to ensure a mutual agreement on the state of the chain and to validate the transactions that have been created on the network. These protocols make sure that all nodes on the BC network arrive at a consensual agreement on the status of the chain and the data it holds. This prevents the repetition of transactions, addition of a malicious node and other security hazards^27^. There are several consensus protocols used in BC as shown in Fig. 4. Table 1 compares the frequently used protocols.

Proof of Work (PoW): In this protocol, the new node has to solve a cryptographic puzzle. It involves finding a nonce corresponding to a hash in line with the network’s criteria. The first node to complete the puzzle joins the network. This protocol is used in Bitcoin and Ethereum 1.0^28^.

Byzantine Fault Tolerance (BFT): This protocol is derived from the byzantine generals problem. It allows consensus to happen even in the presence of malicious nodes. If more than 50% of the nodes agree to a particular event or transaction, then that particular transaction or event is allowed to happen. Used in Neo and Tendermint.

Proof of Stake (PoS): The nodes hold a stake of the currency or tokens used in the BC network. The greater the stake, the higher the chances of getting picked. They are selected randomly (based on the value of their stake) to authenticate the transactions and append blocks to the chain. Mostly used in Cardano and Ethereum.

**Table 1:** The merits and demerits of various consensus protocols.

Consensus protocol	Merits	Demerits
PoW	High security, widespread usage and decentralised	Uses a lot of energy, mining power can be used to centralise the system
PoS	Energy efficient, faster than PoW	Not widespread, stakeholder monopoly might be possible
DPoS	Increases scalability, is fast, Efficient	Can be monopolised and centralised
BFT	Resistant to malicious nodes, the time taken to reach consensus is shorter than that of PoW or PoS, improves scalability	complexity, requires more resources, and is susceptible to centralization.

Delegated Proof of Stake (DPoS): In this protocol, users can choose the nodes based on their promises to confirm the transactions and add new blocks. It is used in EOS and Steem.

### Smart contract

**Fig. 5:**
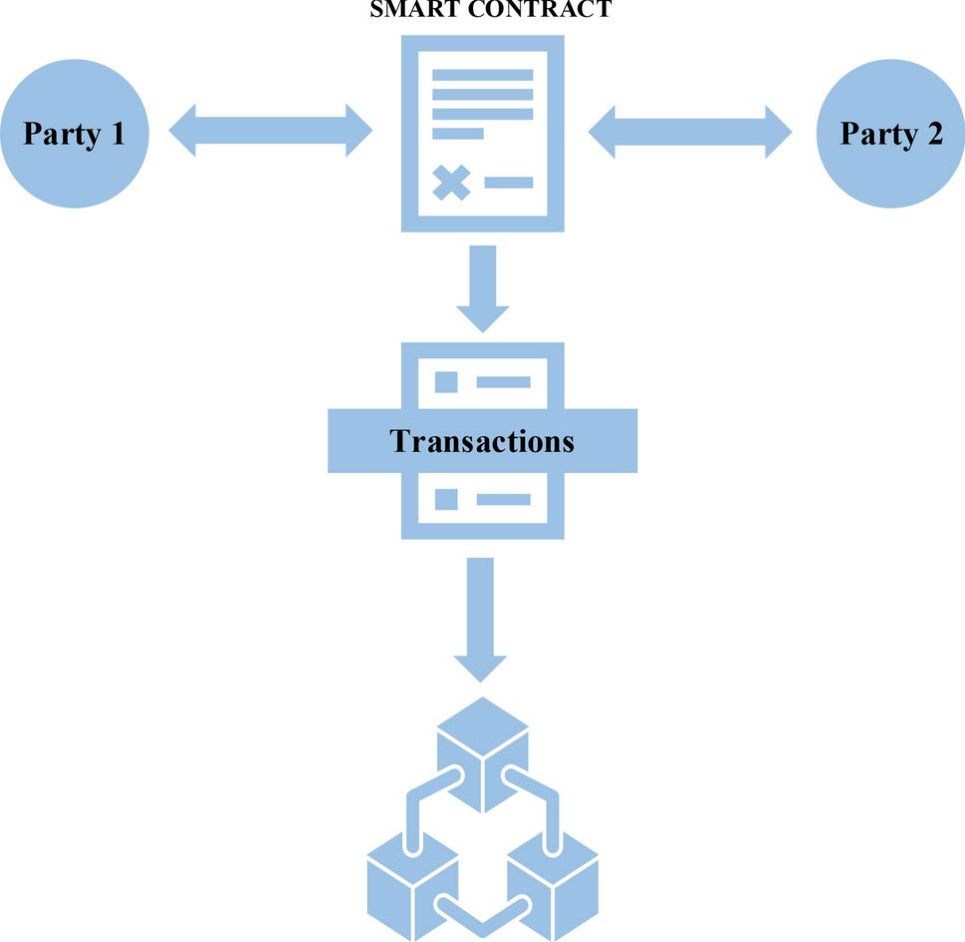
Working of a smart contract.

Any legally binding agreement that sets terms and conditions between two or more parties and defines the relationship between them is called a contract. It must be validated by a court and written in official language. The terms and conditions of a contract are automatically executed in a SC. It is a self-executing electronic code that is stored on the network. SCs are immutable, transparent, and resistant to fraud and tampering. It is generally computer code that runs on the BC network. Unlike traditional contracts, SCs are executed automatically and do not require an intermediary or judiciary. This decreases transaction costs and increases the efficiency of the network. These contracts are available to all network participants and cannot be tampered with at any point. They enforce all the terms and conditions regarding payment and asset transfer agreed upon by all the participants^29–32^. Figure 5. depicts the working of a SC. Once the terms of the contract are satisfied by both parties, the transaction is automatically executed and then stored on the BC ledger.

SCs function on decentralised platforms like Ethereum, utilising BC to record and authenticate transactions. They are most commonly employed in situations that require high standards of trust and transparency, like in financial transactions, supply chain management, and legal agreements. One notable use case of the SC is in the financial sector, in decentralised finance (DeFi). The SCs facilitate the creation of automatic borrowing and lending protocols and help tokenize the assets and store all the data in the BC, thereby obivating the need for intermediaries, streamlining the process, and reducing costs. In supply chain management, SCs can be utilised to enhance traceability, accountability, and automated payment upon successful delivery of goods^33–37^. In the energy sector, BC, along with SCs, is the best solution for P2P energy trading and also to decentralise the entire energy landscape. BC can be used to secure the green hydrogen supply chain and to issue and secure RECs. By programming these contracts to execute clauses in response to predefined conditions, ambiguity and the possibility of disputes are reduced. The code and execution history of a deployed SC cannot be modified, hence providing a secure and tamper-proof ledger of transactions.

## Microgrid

**Fig. 6:**
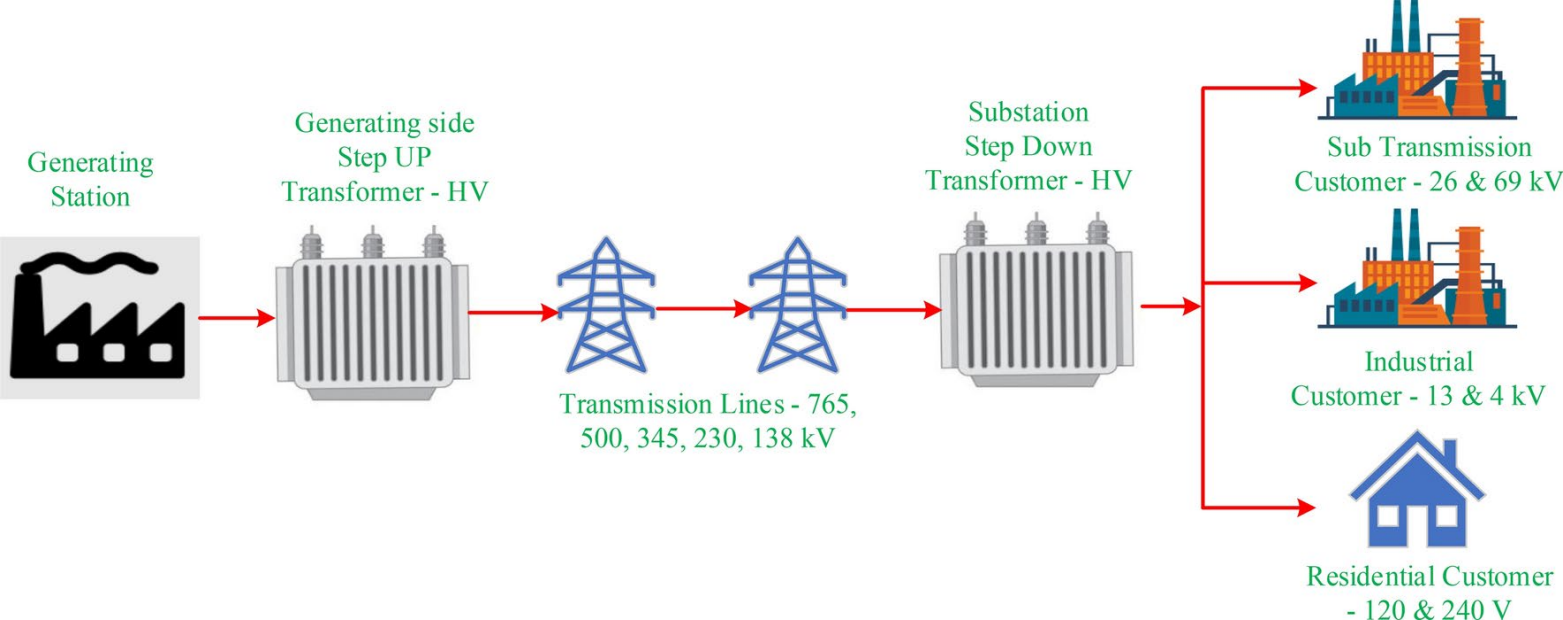
Conventional energy grid.

Traditional infrastructure has been used for the generation, transmission, and dispensation of power conventionally. The conventional, or traditional, grid is a centralised system that has been in place for many years in many countries all over the world. These grids rely on centralised power generation facilities that use fossil fuels, such as thermal and nuclear power stations^38^. To meet the energy demand, conventional power plants generate power on a large scale. This power is then transmitted through a network of high-voltage (HV) transmission lines over long distances to distribution networks or substations. These substations and distribution networks distribute electricity to end users such as industries, commercial users, and domestic users through transformers, substations, and distribution lines, as shown in Fig. 6. The power flow in these types of grids is unidirectional-only from the grid to the consumer and not vice versa^39–43^. The end user is typically not involved in the management or decision-making related to the management of these grids by the local and central authorities. These grids are designed to maintain stability and can withstand disturbances such as voltage fluctuations, equipment failures, or natural disasters. Various control mechanisms and protective devices are in place to manage grid reliability and minimise disruptions.

**Fig. 7:**
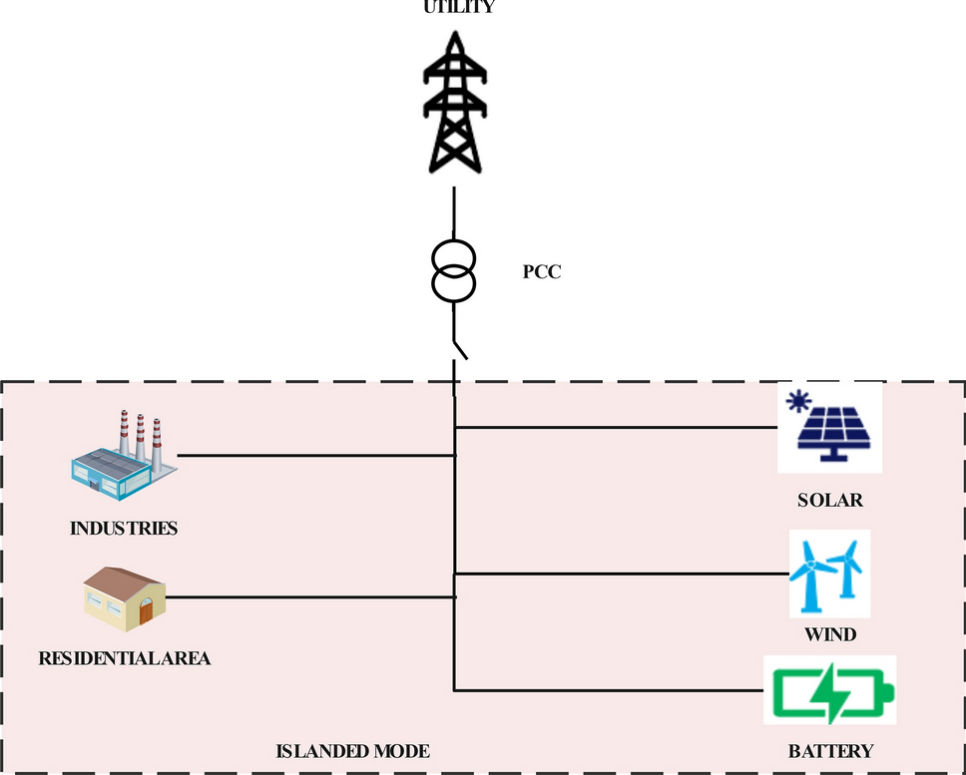
Microgrid.

Conventional grids often do not have dynamic control over several grid parameters, like voltage, load, and power quality, at the consumer level. These grids often have either a predicted value or an average value of these parameters. This poses serious challenges to the functioning of the grid and affects its performance^44^. Upgrading the conventional grid to meet the ever-increasing demand and to integrate RE sources is challenging regarding the development of infrastructure, investment, and regulatory framework. The conventional grid is vulnerable to failures and disruptions like equipment malfunctions, cyberattacks, and natural disasters. Failure at just a single point can cause widespread power outages, leading to economic losses and public inconvenience^45^.

Traditional grids also suffer from power quality issues, including voltage fluctuations, frequency variations, and harmonic distortions. These issues affect the performance and lifespan of electrical equipment and appliances, thus leading to inefficiencies and increased maintenance costs. In a traditional grid, control and decision-making authority are typically centralised with the utility or grid operator^46^. This approach limits the optimisation of generated power and power consumption based on local demands and conditions. Traditional grids heavily rely on fossil-fuel-based power generation like thermal and natural gas power plants, thereby contributing heavily to air pollution, greenhouse gas emissions, and environmental degradation. Most of these grids lack the flexibility to accommodate distributed energy resources (DER’s) because of regulatory barriers and a lack of infrastructure. These grids often face challenges with transmission and distribution losses, particularly over long distances. Energy is lost as heat during transmission and distribution, resulting in reduced overall efficiency and higher costs. These disadvantages have a detrimental effect on the grid, its efficiency, and the quality of power produced and transmitted to consumers. To overcome these disadvantages, the shift to microgrids and smart grids has been initiated by many countries world-wide^47–50^.

A microgrid (see Fig. 7) is a self-sufficient, localised energy system that consists of DERs, power generation sources, and all loads that are interconnected within a well-defined boundary and operates either independently or along with the main power grid. A microgrid has two modes of working: islanded mode and grid-connected mode. The microgrid is disconnected from the main or utility grid in islanded mode. In this mode, providing constant voltage at a stable frequency and with synchronisation between all the DGs (distributed generators) in the microgrid is a big challenge^51–53^. In grid-connected mode, there is a connection between the microgrid and the utility or main grid. The utility grid maintains the voltage and frequency, and in the event of a failure or power outage, it can decouple from the utility at the point of common coupling (PCC). A microgrid has several key factors:

1. Localised power generation: Microgrids have their own power generation sources, such as solar, wind, hydroelectric generators, etc. These generating sources are often located on the distribution side and very close to the load.
2. Energy storage: Energy-storing technologies like flywheels or batteries can be incorporated in the microgrid to store the excess electricity generated during low-demand periods. This stored energy can be used when demand is high or in cases of outages or reduced RE generation.
3. Control and management system: This system manages the power flow within the grid. This ensures that the demand-supply balance is well maintained. It also coordinates the various generation sources, their operations, manages the energy storage and controls the interaction of utility grid with the main grid.
4. Islanding capability: As mentioned above, microgrid can operate independently in case of power outages and emergencies. This ensures that the power supply to critical loads within the microgrid is maintained without any fluctuations.
5. Grid interconnection: While microgrids are allowed to operate autonomously, they can also connect to the main grid. This facilitates the exchange of electricity between the microgrid and the utility, enabling the import or export of energy as needed. Grid-connected microgrids can also provide ancillary services to support the main grid’s stability and reliability^54,55^.
6. Load management: Microgrids serve local loads, which can include residential, commercial, or industrial consumers. Load management within a microgrid involves optimising energy consumption, DR strategies, and load shedding during periods of high demand or system stress.
7. Resilience and reliability: Microgrids enhance grid resilience and reliability by providing localised power supply, reducing the impact of grid disturbances, and enabling faster restoration during outages. Microgrids can also improve power quality and minimise losses associated with long-distance transmission.
8. RE integration: Microgrids are often designed to integrate RE sources, thereby reducing reliance on fossil fuels. The amalgamation of RE, energy storage, and intelligent control systems allows microgrids to optimise the use of green and clean electricity and reduce greenhouse gas emissions.

**Table 2:** Comparison of a traditional grid, microgrid and a smart grid.

Characteristics	Conventional grid	Microgrid	Smart grid
Size and scope	Large-scale, centralised	Localised	Regional and interconnected
Control	centralised control	Localised control	decentralised and dynamic
Energy sources	Limited renewables	Mix of sources	Diverse, including renewables
Autonomy	No autonomy	Partial autonomy	High autonomy
Resilience	Vulnerable to outages	Increased resilience	Enhanced resilience
Reliability	Moderate reliability	Variable reliability	Improved reliability
Flexibility	Limited flexibility	Moderate flexibility	High flexibility
Energy storage	Limited or none	Often integrated	Integral part
Communication	Basic communication	Limited communication	Advanced communication
Efficiency	Moderate efficiency	Variable efficiency	Improved efficiency
Response to outages	Slow recovery	Faster recovery	Rapid recovery
Integration of renewables	Limited integration	Some integration	Extensive integration
Grid management	Basic monitoring	Localised management	Advanced monitoring, control
Load management	Limited DR	Basic load management	Advanced DR
Environmental impact	Higher emissions	Variable impact	Lower emissions
Scalability	Limited scalability	Limited scalability	Highly scalable

## Smart grid

Microgrids can also be upgraded with communication protocols and automation to acquire real-time grid data by making them smart grids. Unlike the conventional grid, smart grids have a host of networks of technologies that facilitate real-time communication, the exchange of data, and real-time control across most of its components. It offers a wide variety of multi-dimensional advantages and benefits for the energy ecosystem, like real-time communication, immediate fault correction, accuracy, and reliability of data. The foundation of a smart grid is advanced metering infrastructure (AMI), which enables bidirectional communication between users and utilities, real time data collection, DR, and accurate billing^56–59^.

In the distribution automation phase, smart grid employs intelligent devices, sensors, communication devices, and networks to manage and monitor the flow of electricity and to identify and rectify faults immediately to bolster grid reliability^60^. The use of energy storage technologies balances the load and integrates RE easily. The smart substations in this grid use high-voltage direct current (HVDC) systems and phasor measurement units (PMU) to ensure optimal power flow, voltage control, and seamless integration of RE resources. A microgrid is a localised power system that consists of DERs, energy storage, and loads, operating either along with the main grid or autonomously in the case of grid failure. Unlike microgrids, smart grids cater to much broader geographic regions, like the distribution and transmission infrastructure of the entire utility grid, and cannot operate without the grid^61–66^. Smart grids excel in large-scale energy management, enhancement of reliability, and regional DR. Whereas the microgrid provides localised resilience and addresses energy needs within a specific community, like a campus or industry, and that too mostly during power outages (Table 2).

**Fig. 8:**
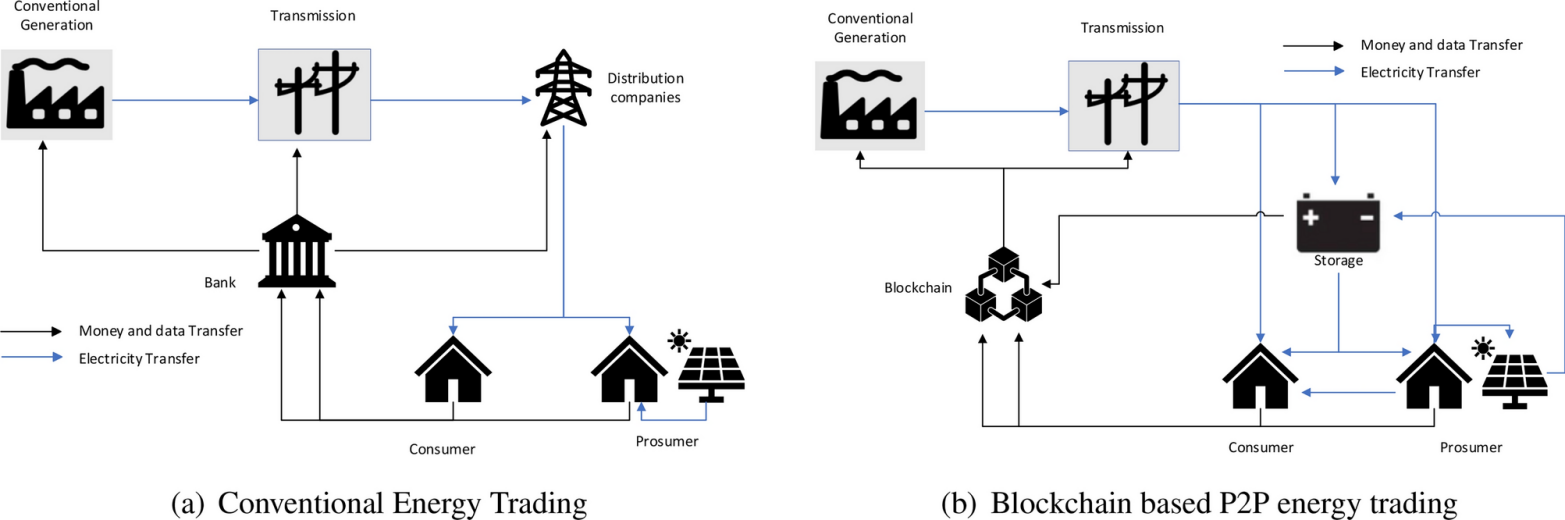
Energy market scenario.

**Table 3:** The existing projects on energy trading using blockchain and their key features.

Project name	No of transactions(until feb-2024)	Energy efficiency	Data privacy and security	Interoperability	Governance model	Technological innovation	Regulatory compliance	Real-world adoption and impact
Power ledger	> 783,974	Moderate	Robust security measures	Interoperable with some platforms	decentralised governance model	SC utilisation	Compliant with local energy regulations	Widely adopted in several countries, facilitating significant energy trades
We power	> 205915	High	Strong data encryption	Interoperable with existing energy infrastructure	Hybrid governance model with community involvement	Integration with IoT devices	Compliant with energy market regulations	Gaining traction, with notable RE projects using the platform
Grid+	47,655	High	Privacy-focused design	Limited interoperability with other platforms	DAO (decentralised Autonomous Organisation) model	Advanced data analytics and AI integration	Compliant with local and regional energy regulations	Pilot projects underway, demonstrating positive impact on energy costs
Electron	36, 079, 198	Moderate	BC-based data encryption	Interoperable with selected energy markets	Consortium governance model with industry stakeholders	Peer-to-peer energy balancing and optimisation algorithms	Compliant with energy market regulations	Successful implementation in a few local energy communities
LO3 Energy	–	Moderate	Immutable transaction records	Interoperable with compatible energy systems	Consensus-driven governance model	Microgrid optimisation algorithms	Compliant with energy market regulations	Pilots conducted, showing potential for localised energy trading
Sun Contract	40,131	Moderate	Privacy-centric protocols	Interoperable with some energy providers	decentralised governance model with community voting	Integration with decentralised IoT devices	Compliant with energy market regulations	Growing user base, significant energy trades facilitated in specific regions
Electrify	188,824	High	Data encryption and user privacy measures	Interoperable with the national energy grid	Hybrid governance model with industry partners and community involvement	AI-based DR algorithms	Compliant with energy market regulations	Notable adoption in a specific market segment, showcasing cost savings
Greeneum	–	Moderate	Secure data transmission	Interoperable with selected energy systems	Consortium governance model with participating energy producers	Predictive analytics and machine learning for energy trading	Compliant with energy market regulations	Ongoing trials with positive feedback from participating energy providers

## Applications of blockchain in the energy sector

BC technology has the potential to transform the way electricity is generated, distributed, and consumed because of its decentralised and transparent nature. It can also verify and record the transactions securely and address all the key challenges faced by the energy sector, like the integration of RE, efficient energy trading, grid management and much more^67–72^. It can facilitate P2P energy trading seamlessly and thus help integrate RE into the grid efficiently. BC can also be used in emerging energy technologies like green hydrogen. It can be deployed in the green hydrogen supply chain or to connect all green hydrogen charging stations in the future. It can also be used to integrate Internet of Things (IoT) devices and electric vehicles (EVs). By using BC, the energy sector can become a more sustainable and decentralised energy ecosystem^73–75^.

### Energy trading

One of the main uses of BC in the energy landscape is decentralised energy trading. Traditionally, in the conventional grid (Fig. 8a), energy trading was a centralised process, with banks and energy companies acting as intermediaries. The distributing companies are the central authority related to the electricity transfer in this trading method. These companies get electricity from main grid, and transfer it to the end user, even without having any idea of the local demand and supply scenario. The banks are the central authority for all the monetary transactions, and it takes an exceptionally long time to verify these transactions. These transactions happen with the bank acting as a central authority. This increases the cost of electricity and most of the time, the intermediaries have no idea about the local demand and supply scenario^76–81^.

However, using the BC, as shown in Fig. 8b enables the creation of P2P electricity marketplaces, allowing consumers to directly trade excess energy with one another. Through SCs, transactions can be automatically executed, thus ensuring safe and transparent energy trading and eliminating the need for third-party authentication. This decentralised approach empowers consumers by giving them more control over their energy choices and also promotes the utilisation of DERs such as solar panels or wind turbines. With the exchange of energy tokens, monetary transactions can easily be verified by a SC and the need for banks can be eliminated, thus saving a lot of time^82–87^. Using smart meters, and BC gives real-time data on the energy available with each prosumer and consumer, i.e., the amount of energy needed by the consumer and the energy available with the prosumer.

**Table 4:** The existing literature on energy trading using blockchain.

Paper	Platform of blockchain used	Key aspects	Discoveries	Merits	Demerits
^43^	Various BC types are reviewed, including public and private BCs.	Provides a comprehensive review of BC-based P2P energy trading, discussing various technical and non-technical aspects.	A review of the types of BCs used in energy trading, the challenges faced, and the types of trading were discussed	Increased efficiency, transparency, and autonomy in energy trading.	Challenges in scalability, privacy, and regulatory compliance.
^77^	Ethereum	Proposes a decentralised P2P trading by deploying BC and SCs.	Demonstrates the feasibility of using BC for secure and transparent energy trading.	Reduced reliance on intermediaries, increased transparency, and improved energy efficiency.	Scalability issues with Ethereum BC.
^78^	Ethereum	Presents a BC-enabled P2P energy trading framework, considering RE sources.	Shows the potential of BC for enabling secure and efficient P2P energy trading.	Increased transparency, reduced costs, and optimised energy utilisation.	Scalability limitations and energy market regulatory challenges.
^41^	Hyperledger fabric	Proposes a BC-based P2P trading design using Hyperledger Fabric.	Demonstrates the applicability of Hyperledger Fabric for secure and efficient energy trading.	Enhanced security, privacy, and flexibility in energy trading.	Complexity in setting up and managing the Hyperledger Fabric network.
^79^	Ethereum	Presents a BC-based P2P energy trading framework considering microgrid characteristics.	Demonstrates the potential of BC in enabling secure and decentralised energy trading in microgrids.	Improved transparency, reduced costs, and increased grid resiliency.	Scalability challenges and reliance on third-party energy aggregators.

Various BC platforms with different consensus protocols can be used to facilitate trustworthy and secure transactions in the grid, since BC greatly amplifies security, transparency, and efficiency. Regarding energy trading in microgrids, the main use case of BC is P2P trading, which enables direct transactions between consumers and prosumers. This process bypasses centralised intermediaries such as utilities and banks, thereby reducing transaction costs, increasing autonomy in energy trading, potentially optimising the energy usage of network participants, and also enhancing the overall efficiency of the system. It can also promote the integration of RE sources by allowing more direct and localised trading of RECs within microgrids^88–98^. There are various companies and trading platforms for energy trading using BC. Some of the projects and their key features have been compared in Table 3 and the other existing literature regarding BC-based energy trading is given in Table 4.

PowerLedger uses a customised and permissioned BC called the Solana BC. This utilises proof-of-history and PoS consensus protocols and is less energy-intensive compared to the other PoW BCs. This BC has been implemented in various projects all over the world. Using the xGrid P2P technology, PowerLedger partnered with the ISGF and the Uttar Pradesh government to create a pilot project for energy trading using BC. With a 27 GW total installed capacity, the P2P market buy price was 43% lower than the retail electricity price for the same quantity of electricity. In Delhi, PowerLedger partnered with Tata Power-DDL to facilitate P2P trading of solar power between 150 metres in North Delhi. And in Calcutta, with the partnership of ISGF and Calcutta Electricity Supply Corporation (CESC), it provided for P2P trading between over 1000 consumers (213 prosumers and 788 consumers) and saved 233,703.73 INR in 5 months. In Australia, this company has implemented two pilot projects. East Village Project (EVP), sponsored by the Australian government, and the Evermore Project. In EVP, 36 homes with rooftop solar panels were facilitated to trade with each other, and achieved an 80% reduction in grid energy usage. By using a 56.4 kW solar and a 156 kWh battery hybrid system for 24 apartments and a common area in evermore, PowerLedger has achieved a milestone of 430 kWh of RE traded per week, $540 savings per household annually, and a 36.9 tonne reduction in CO_2_ emissions.

**Fig. 9:**
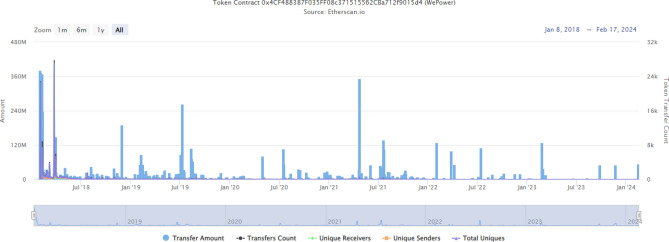
Transactions done using the WePower smart contract^99^.

In Estonia, WE Power, an energy trading company, implemented a BC-based smart energy token system. They uploaded 26,000 h and the aggregated consumption and production data of 24 terawatt hours into the BC and converted them into 39 billion smart energy tokens, with each token representing a kWh of power. This project involves the participation of 26,000 nodes and over 205,915 transactions from the time of inception, as shown in Fig. 9. SunContract, another energy trading platform, has more than 10,000 customers in Slovenia. They use a hybrid PoS and Proof-of-Authority (PoA) consensus mechanism and have over 78 active nodes on the Mainnet Explorer and 46 active nodes on the Testnet Explorer. Lo3 Energy uses their custom-made BC called EXERGY to facilitate P2P trading between various nodes in the Brooklyn microgrid. They have also implemented this Cornwall local energy market by partnering with Centrica and serving over 21.8 million customers in 2018. They have deployed over 40 e-meters and have already been contracted to install 275 more metres.

In partnership with London Hydro, Electron has developed a BC-based electricity trading project to ensure the visibility of DER’s and to make sure the locals fully utilise the DER’s. With a participation rate of 40%, a total of 5 DR events took place in a span of 5 months. Electron also partnered with Silicon Valley Clean Energy to design an energy marketplace to incentivize load reduction and managed to achieve a reduction in CO_2_ emissions of 250,000 million tonnes per year. In Scotland, they have developed a local real-time flexible energy marketplace and allowed the local wind generators to trade with local consumers during the windy period. In a span of one year, over 23,600 trades were completed, and 8.26 MWh of energy was dispatched.

However, there are several techno-legal challenges associated with the implementation of BC. Regarding P2P energy trading, BC encounters significant obstacles, the most prominent of which are scalability concerns, especially with Ethereum, which have been identified as a limitation that needs to be addressed for widespread adoption. As the number of nodes and the volume of transactions escalate, certain BC networks might encounter difficulties in maintaining adequate processing speeds and capacity. Consequently, this can result in transaction delays and increased costs. These transaction delays, also known as transaction latency, are not suitable for P2P energy trading, where simultaneous communication and energy transfer need to happen. Also, the energy consumption of PoW blockchains is immense due to the high computational power required. This high energy consumption is unsustainable and contradicts the very purpose of P2P energy trading. Among the technical challenges, one prevalent challenge that arises is the absence of interoperability among diverse BC platforms. The fact that ongoing initiatives have been developed on distinct BC networks may impede their ability to share and interact seamlessly. The absence of uniformity impedes the ability of diverse P2P energy trading initiatives to operate in concert. Another significant challenge is the fragmentation of the energy market. The energy market is frequently fragmented, with distinct regulatory frameworks and market structures in each region^100–105^. Pre-existing initiatives might encounter difficulties in managing this fragmentation, which could restrict their capacity to expand and establish a unified, interconnected framework for P2P trading. Regulatory uncertainties present an additional challenge, given that current frameworks might not sufficiently consider the distinctive characteristics of decentralised energy trading platforms. The complex process of integrating BC technology into well-established energy infrastructures necessitates significant modifications to conventional models^106–108^. Furthermore, the establishment of consumer confidence and the cultivation of awareness regarding decentralised systems may pose obstacles to user adoption and education.

In the near future, scalability and latency issues can be addressed by the development of consensus mechanisms like proof-of-stake and layer-2 scaling solutions that enhance the efficiency of BC networks. Establishing well-defined and supporting regulatory frameworks is essential for the future widespread adoption of BC in P2P energy trading^109–113^. These frameworks must be adaptable to the decentralised nature of these platforms. The industry’s standardisation initiatives will improve interoperability, promoting the development of a unified and integrated decentralised energy trading environment.

Advanced SCs can have a crucial impact in the future by providing functionalities such as dynamic pricing, automated dispute resolution, and conditional agreements to improve the user experience in P2P energy trading. Enhanced educational and awareness initiatives are crucial for surmounting obstacles in user adoption, facilitating comprehension of BC technology, and fostering trust among end-users^114–118^.

### Green hydrogen supply chain

The ongoing pursuit of clean and sustainable energy solutions has brought renewed interest to hydrogen, a versatile fuel with zero tailpipe emissions when used in fuel cells. While the concept of hydrogen as an energy carrier is not novel, its production using RE sources, known as green hydrogen, presents a potentially trans-formative solution to the global climate crisis. Traditional fuels, like gasoline, release significant greenhouse gases upon combustion, contributing to climate change. Hydrogen, however, reacts with oxygen to produce only water vapour, making it a zero-emission fuel^119–121^. This inherent cleanliness makes it a critical component to achieve net-zero emissions goals and mitigate climate change. Additionally, its high energy density per unit weight compared to gasoline enables longer driving ranges for vehicles and efficient energy storage solutions. This versatility extends beyond transportation, making it suitable for powering turbines for RE storage and replacing fossil fuels in industrial processes. Furthermore, its potential for domestic production could enhance energy security by reducing reliance on imported fuels and turning heavily polluting industries into zero-carbon emission ones. Storing hydrogen for future usage is relatively easier compared to electricity storage, and it offers a potential of hundreds of TWh (over 10,000 times the global electricity storage capacity in batteries). Despite its promise, challenges remain. Currently, producing green hydrogen is more expensive than traditional fuels due to the higher cost of RE and electrolysis technology. Infrastructure for hydrogen refuelling, especially for vehicles, is still in its early stages, hindering widespread adoption. Technical hurdles exist regarding storage and transportation, requiring specialised technology and infrastructure development^122–124^. However, recognising the significance of green hydrogen, investments are surging. Government policies, technological advancements, and growing demand for clean energy solutions are fueling this growth. The international RE agency (IRENA) estimates that by 2050, green hydrogen could meet 25% of global energy demand, highlighting its potential to transform our energy landscape. The Indian government aims to produce 5 million mt of green hydrogen per year by 2030 and to reach an electrolysis capacity of 15 GW by the same time-frame. This ambitious target would enable the country to reduce greenhouse gas emissions by 50 MMT and decrease fossil fuel imports by over 11.4 billion euros.

Unlike its counterparts, like grey hydrogen, which is produced from natural gas and releases significant CO_2_ into the atmosphere, and blue hydrogen (similar to grey hydrogen but capturing some CO_2_ emissions), green hydrogen is truly sustainable. No net CO_2_ emissions are released during its production or use, making it a critical weapon in the fight against climate change. While cost and production limitations persist, significant efforts are underway to address them. The projected decrease in green hydrogen cost from €2.5 to €6/kg of $H_2$ currently to €1–€2/kg of $H_2$ by 2030 paints a promising picture. Further, investments, expected to reach €170 billion by 2030, demonstrate the collective commitment to unlocking this technology’s potential^125–127^.

**Fig. 10:**
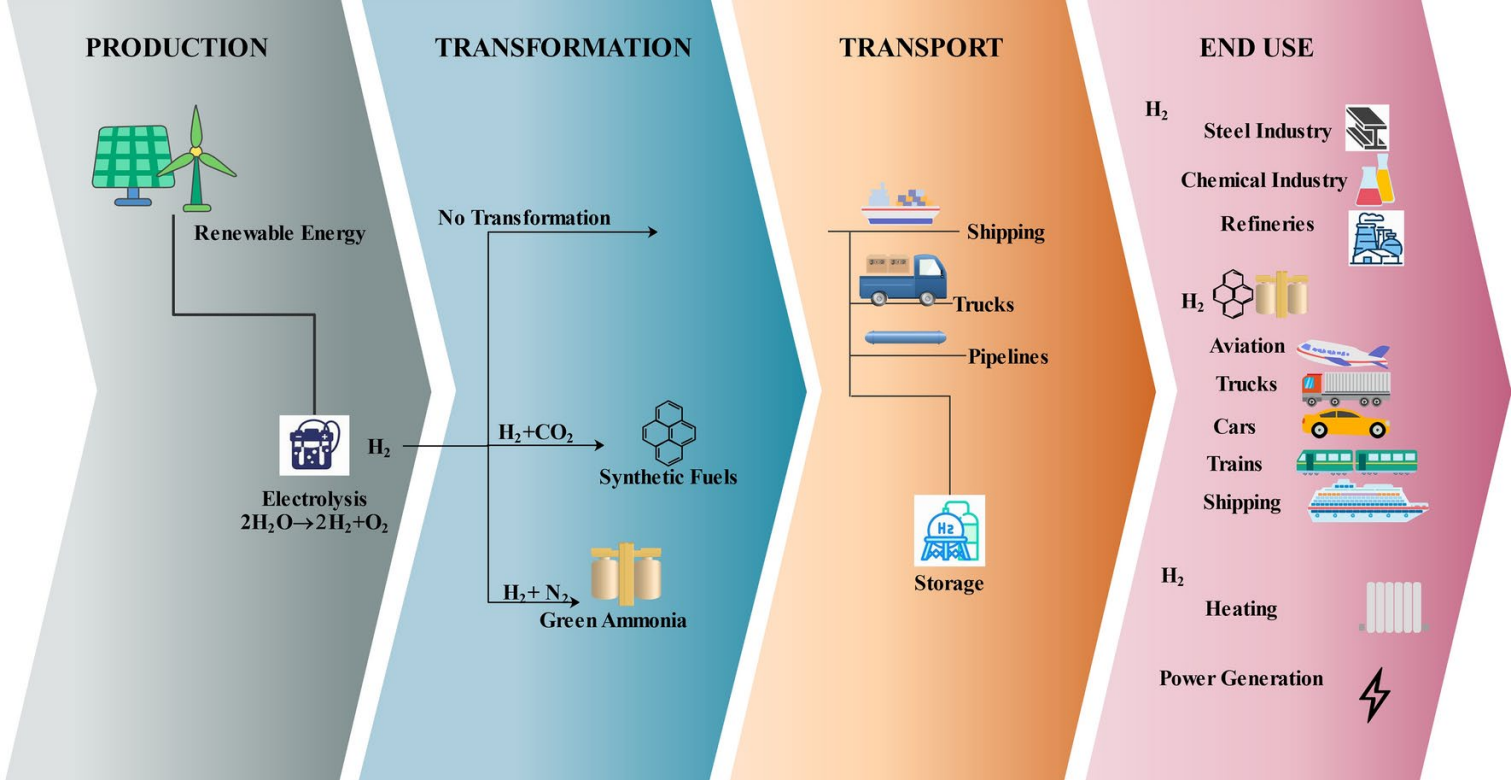
Various stages of a green hydrogen supply chain.

Within the energy industry, green hydrogen is causing a stir since it is hailed as a crucial participant in the shift to sustainable energy. As shown in Fig. 10, the utilisation, distribution, and production of this item constitute a complex supply chain that can greatly benefit from BC technology. Green hydrogen is generated via the electrolysis process, which uses electricity generated by RE sources like solar or wind to divide water into hydrogen and oxygen. This environmentally friendly and sustainable method of producing hydrogen is integral to the green energy ecosystem. There are multiple phases in the green hydrogen supply chain, including production, transportation, and utilisation. BC technology, with its decentralised and immutable nature, is a transformative solution to address the challenges associated with green hydrogen supply chain management^128,129^. There are various benefits to using BC in the green hydrogen supply chain.

#### Traceability and transparency

They are two of the most significant benefits that BC affords in the renewable hydrogen supply chain. From production to distribution, BC generates a decentralised and public ledger that logs each transaction and movement of green hydrogen. By maintaining transparency, every participant in the supply chain is furnished with up-to-date and precise information pertaining to the source, quality, and carbon emissions of green hydrogen. The integration of BC technology enables stakeholders-consumers, distributors, and producers-to trace the complete trajectory of green hydrogen, thereby validating its manufacturing procedures and guaranteeing adherence to environmental regulations. Hence fostering confidence among participants and advancing the cause for a responsible and sustainable supply chain.

#### Tracking of carbon footprint

Green hydrogen is well acclaimed for its low carbon footprint and environmentally friendly production in comparison to traditional methods of hydrogen production. To track this carbon footprint all along the chain and validate the associated emissions, BC technology is indispensable. BC makes the carbon footprint data tamper-proof and readily accessible for verification since each and every transaction in all the stages is recorded in an immutable ledger. Hence, by deploying BC, both regulatory bodies and consumers are empowered to render well-informed judgements grounded in reliable and up-to-date data concerning the ecological ramifications of green hydrogen.

#### Secure data sharing and collaboration

In a complex supply chain with several stakeholders and huge volumes of data, transactions must be protected and executed efficiently. BC facilitates secure data sharing, thereby preserving data integrity and privacy. This is done by granting a permissioned view of the BC to each participant, enabling them to access relevant information while protecting sensitive data. Hence, fostering information exchange and collaboration among stakeholders, and enhancing the efficiency and connectivity of the green hydrogen supply chain. BC also enables seamless collaboration among producers, distributors, and consumers, enabling them to exchange vital data and insights to optimise processes and improve sustainability.

There are various implementations of BC in the green hydrogen industry. HyDeal, a hydrogen ecosystem company, uses Hyperledger Fabric to enable secure data exchange between hydrogen producers, transporters, and end-users. HyDeal Ambition, a project developed by HyDeal, is the largest green hydrogen project with a scale of 3.6 megatons of green hydrogen produced by 95 gigawatts (GW) of solar energy and 67 GW of electrolyser. Another project, HyDeal Espana, is in the pipeline and is expected to start operations in 2028. Poised as the first mass-scale hydrogen hub, it is expected to supply 3 million tonnes of green hydrogen over a span of 20 years to various H2 consumers in Spain at a competitive price compared to fossil fuels. The production capacity for this project is projected to be around 4.8 GW of solar energy and 3.3 GW of electrolysers. In^130^, a BC-based SC system is proposed to automate trade and guarantee the traceability of the hydrogen supply chain.

A sudden increase in demand for green hydrogen could potentially strain the system, resulting in slower transactions and higher costs. Including BC into the current supply chain systems requires significant technical adjustments and these adjustments could potentially face opposition from stakeholders accustomed to conventional approaches. However, standard data formats and protocols are required to ensure seamless exchange of information and data in the supply chain. In addition, the energy consumption of certain BC networks, especially those using the PoW consensus protocol, is very high and raises environmental concerns. This lack of energy efficiency is against the main objective of the green hydrogen sector : sustainability. Hence, more research is required in order to make the BC more energy efficient by using other consensus protocols. Also, there is no standard green hydrogen certification format accepted worldwide. A vigorous certification process is required to safeguard the integrity of the supply chain and to simplify the tracking and verification processes.

In addition to the above challenges, regulatory uncertainties might hinder the integration of BC into the green hydrogen supply chain. To overcome these challenges, a lot of research is needed in the future, thereby significantly impacting the management of green hydrogen supply chains. The interdependence of green hydrogen supply chains within the whole energy system should be explored to include green hydrogen into the energy sector and not just as a fuel. BC provides smooth integration among different RE sources, resulting in a more inclusive and integrated network for the management of green energy. IoT devices can also be combined with BC technology to improve monitoring and data collection in real time. This can be done by adding smart sensors and devices that send data directly to the BC, which improves the accuracy and speed of data about the production, transport, and storage of green hydrogen.

### Real-time demand response

The demand for electricity has increased as a result of population growth and technological advancements, which conventional power systems cannot meet on their own. To bridge this demand-supply gap and generate electricity through renewable energy, microgrids are being employed. However, this alone is not enough to bridge the huge gaps between demand and supply and maintain grid stability, and hence DR mechanisms are used. DR is a critical tool that enables microgrid operators to optimise energy utilisation and enhance grid reliability. DR refers to programmes that encourage consumers to modify their electricity consumption patterns in response to grid conditions or pricing signals. In conventional systems, utilities adjusted generated power to close the demand-supply gap. Contrary to the traditional method, DR empowers consumers to become active participants in grid management. Users can choose from a variety of DR mechanisms, like price-based DR and direct load control mechanisms. In the direct load control programme, utilities can control specific user appliances based on user consent. Whereas in price-based DR programmes, lower prices are offered during off-peak hours to incentivize users to shift non-critical loads (e.g., running dishwashers or laundry machines) to these periods and higher prices during peak demand times to ensure that the users reduce consumption or use alternative energy sources (e.g., battery storage in homes)^131,132^.

Employing DR in microgrids offers various advantages, like enhancing the efficiency of energy utilisation within the microgrid. It also reduces the dependence on expensive and polluting power plants by encouraging users to shift consumption away from peak periods. It also helps consumers and operators save money. Consumers benefit from lower energy bills during off-peak hours, while the microgrid operator experiences reduced operational costs by minimising reliance on expensive peak power. DR programmes significantly improve grid reliability by actively managing demand fluctuations. DR programmes also help maintain grid stability and mitigate the possibility of blackouts, particularly in microgrids with high penetration of variable RE sources. However, the implementation of effective DR programmes in microgrids faces several challenges. The main challenge lies in securing active participation from consumers. Although some users might be incentivized to join the DR programme, convincing others to change the consumption habits they are accustomed to requires targeted outreach and education programmes. Additionally, collecting and managing consumer energy data for DR purposes requires robust cybersecurity measures and clear data ownership protocols to ensure user trust. To effectively manage real-time data and dispatch appropriate DR signals, advanced communication infrastructure and sophisticated algorithms are required.

These challenges can be tackled by emerging technologies like BC. By employing BC, a secure and transparent record of energy consumption and associated incentives can be stored immutably, thereby fostering trust and encouraging broader consumer participation. This eliminates the need for a centralised repository of consumer data and further bolsters data security and privacy. Using BC, SCs can also be employed to automate the entire DR programme and streamline the entire process. SCs can be made to automatically trigger DR actions based on predefined conditions, such as price fluctuations or grid congestion, thereby eliminating centralised control and the need for intermediaries^133–135^. Since the BC is immutable, the data contained in the BC cannot be tampered with. This increases trust in the process and prevents malicious tampering with data. Thus, the core attributes of BC, such as immutability, transparency, and decentralisation, significantly enhance the efficiency of DR programmes^136–139^.

**Table 5:** Comparison of the existing literature on demand response using blockchain.

Paper	Key focus area	Methodology	Type of BC	Contributions
^139^	DR in microgrids	Proposed system design and simulation.	Not specified	Introduced a BC-based DR system.
^138^	decentralised DR in smart grids	Proposed a decentralised architecture	Public BC	Utilised BC technology for decentralised DR
^140^	DR mechanism for microgrids	Proposed BC-based framework	Permissioned BC	Developed a BC-based DR mechanism
^141^	DR framework for energy trading in microgrids	Proposed a BC-based framework	Private BC	Introduced a BC-based framework for energy trading
^142^	SCs for DR in microgrids	Proposed SC implementation	Public BC	Utilised SCs for DR in microgrids

Table 5 shows the existing literature on BC-based DR programmes in microgrids. Grid demand can be efficiently met by fostering greater flexibility among the grid participants by helping them engage in real-time energy transactions.^140^, highlights the benefits of using BC, such as improved transparency, reduction in transaction costs, and enhanced data security. The proposed framework uses SCs to automate the DR process and enable efficient energy trading among microgrid participants^143^. SCs facilitate real-time and automated DR programmes, thereby ensuring streamlined, decentralised decision-making. This approach deals with real-time user data and hence fosters efficient energy trading and enhances the flexibility and responsiveness of microgrids^144–150^. Although leveraging BC offers multiple advantages in DR programmes, there are various challenges to seamless grid operation and simultaneous data transfers. PoW blockchains like bitcoin and ethereum 1.0 struggle with handling high volumes of real-time data associated with DR programmes. DR programmes require the BC to be integrated into the grid seamlessly, for which the BC should be compatible with the security measures, communication protocols, and data formats used in the grid. The current DR programmes and associated regulations need to be adapted to accommodate BC-based solutions and also to address issues such as dispute resolution, data ownership, and liability within a BC-based DR system. Despite the challenges discussed above, ongoing research and implementation efforts are building upon these studies to further explore and refine the integration of BC technology in real-time DR systems. Scalable BC architectures with improved latency and reduced energy consumption are being tested to improve the overall efficiency of the system.

### Renewable energy certificates

The rapid integration of RE sources into power grids necessitates robust mechanisms to incentivize their development and ensure the environmental attributes associated with their generation are properly accounted for. RECs provide a solution to this by ensuring traceability and accountability for each MWh of electricity generated through RE sources. The certification of RECs is a well-defined process. A designated certificate-issuing body makes use of meticulous metering & monitoring systems and verifies the details and attributes of the electricity fed to the grid. After successful verification, one REC is issued for every MWh of RE generated. The REC ownership is electronically registered, and the particular RE source will be linked to it. RECs help create additional revenue streams for prosumers, thereby incentivizing greater participation in RE generation. This, in turn, enhances the development of new RE projects and their economic viability. RECs also help companies meet the renewable purchase obligations (RPOs) imposed by certain governments. In certain countries, companies and organisations are mandated to procure some of their electricity from RE sources. Even if the companies do not meet this requirement, the RPOs can be met by purchasing the equivalent number of RECs. Finally, RECs help maintain grid security and stability and promote diversification of electricity generation, thereby reducing the dependence on fossil fuels.

RECs are vulnerable to duplication or fraud and can potentially compromise the environmental and monetary benefits associated with REC ownership. Insecure RECs can lead to a double-counting problem, which inflates the anticipated environmental benefits of the grid. This erodes trust among market participants, leading to a decline in the development of new RE projects. Therefore, the security and integrity of RECs are important to maintain trust and ensure the effectiveness of the system. These challenges can be overcome by deploying BC to secure REC issuance and management. BC is an immutable and distributed ledger, meaning all network participants have a copy of the transactions, eliminating any possibility of tampering or fraud. All participants in the network can verify the authenticity and ownership of RECs, and when any tampering is made, it can easily be detected due to the hash-linkage in BC. If any tampering is done on a particular node, the hash of that node changes, and this is reflected in the next node. This hashing system of the BC significantly reduces the risk of duplication or manipulation. Secondly, BC ensures transparency throughout the entire REC lifecycle. From initial generation verification to subsequent ownership transfers, all transactions are recorded on the BC and can be verified at any point in time, thereby enhancing trust among market participants. Finally, by leveraging SCs, the certification process can be automated, including key processes like REC issuance and trading. Thus streamlining administrative procedures, reducing transaction costs, and further enhancing the efficiency of the REC market.

**Fig. 11:**
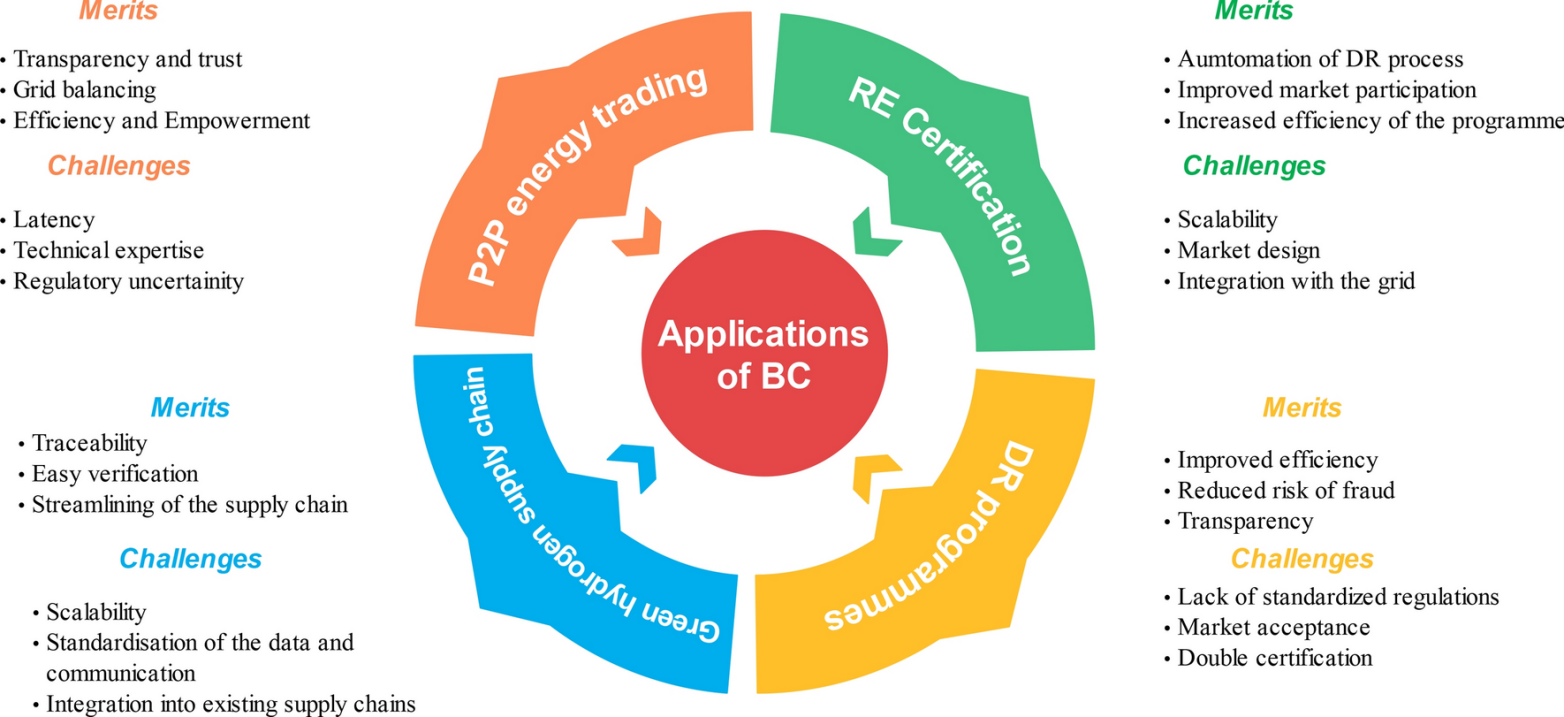
Merits and challenges of the applications of BC in energy sector.

Delardas and Giannos^151^ explores BC-enabled RECs for microgrid applications. This work makes use of SCs on a BC platform to automate and streamline the issuance, trading, and settlement of RECs. It shows that BC can facilitate a more efficient and reliable REC market by eliminating intermediaries and reducing transaction costs. A BC-based P2P energy trading and REC system in microgrids is proposed in^152^. This study advocates for distributed ledger technology that allows direct energy and REC transactions between microgrid participants, promoting a decentralised and transparent marketplace. This paper demonstrates the feasibility and benefits of BC for enabling secure REC trading within microgrids. In^153^, a BC-based REC management system for microgrids is proposed. They emphasise the role of BC in ensuring the integrity and authenticity of RECs by recording each transaction on BC. BC technology can enhance trust and streamline REC management processes, contributing to a more reliable and robust microgrid ecosystem. In^154^, a BC-based REC system specifically designed for microgrids is proposed. This study outlines the assimilation of BC technology with smart meters and IoT devices to authenticate and track RE generation and consumption. By leveraging BC’s transparency and security features, the authors envision an REC system that can accurately allocate and verify RE credits within microgrids. By leveraging decentralised and immutable ledger systems, BC can transform the REC market, empowering microgrid participants to engage in secure and peer-to-peer trading of RECs^155,156^.

Level-1 and PoW blockchains are incapable of handling large amounts of data simultaneously and face scalability issues that lead to a surge in transaction latency and decreased effectiveness of the network. When the data volume rises, the burden on the chain increases, thereby increasing the duration to complete a single transaction. Also, integrating data from all network participants, like RE sources, utilities, and regulatory bodies, is very difficult without standardised data formats and communication protocols. There might also be duplicate RECs issued due to the high volumes of data present in the system. The BC network should be able to prevent this double certification in order to guarantee the robustness of the certificates. These challenges can be overcome by scalable, second-generation and third-generation blockchains. Research is ongoing to develop energy-efficient, interoperable BC solutions. The implementation of BC technology in REC management offers exciting prospects for optimising RE utilisation in microgrids as the energy sector transitions towards a more sustainable future.

Figure 11 shows the consolidated merits and challenges pertaining to each application listed above. Even though there are challenges, they can be addressed through technological interventions like layer-2 BCs. Ethereum 1.0 and bitcoin are generation-1, layer-1 BCs. These can be modified by creating another layer on top of them or changing their consensus protocols. Such a platform is ethereum 2.0, a layer-1, generation-2 BC, with a PoS consensus mechanism unlike the PoW in ethereum 1.0. There is more research going on in implementing layer-2 and generation-3 BCs in the energy market to further augment the merits and overcome the challenges posed by BC.

## Conclusion

This paper has provided a comprehensive analysis of the potential applications and benefits of deploying BC technology in the energy market. The role of BC in the energy sector and the transformation it can bring to key aspects of the sector have been underlined. In addition, we have explained how the transparency and immutability of BC can secure data robustly. We have also highlighted how BC overcomes the challenges faced by the traditional energy market effectively. This paper has also underlined the need for the deployment of BC in key areas like P2P energy trading, the green hydrogen supply chain, real-time DR, and REC. We have compared the existing projects and literature regarding each application. Quantitative details about the ongoing pilot projects in the P2P energy market and the green hydrogen supply chain have also been discussed in the paper.

Furthermore, the paper has also discussed the merits and disadvantages associated with the implementation of BC in each application. The challenges pertaining to the utilisation of BC have also been discussed in detail. It has been observed that the existing BCs face several challenges, including scalability, latency, interoperability between different BCs, and regulatory compliance. Also, integrating BC with the existing energy infrastructure is a big hassle and needs to be addressed for a seamless and successful deployment of BC in the energy market. As a whole, the paper has provided valuable research regarding various applications and the potential of BC technology in the energy sector. The paper offers a comprehensive review of the key aspects, merits, and challenges pertaining to the utilisation of BC in various aspects of the energy sector.

## Future scope

The challenges pertaining to the implementation of BC can be overcome by developing layer-2 BC solutions. These work on consensus algorithms like PoS and DPoS that are more energy efficient. Ethereum has already implemented the PoS consensus algorithm and a layer-3 BC for ethereum is also being built to overcome the scalability and latency issues. These newer BCs are more necessary in the energy sector because of the huge volume of data being handled. In the future, the interoperability and interconnection between different BC platforms and the integration of BC into the existing energy landscape of developing countries should be addressed to effectively make use of this technology to benefit humankind. Also, the integration of DR, P2P trading, and REC can be facilitated to enhance the true sustainability of the energy market. From the government side, regulations need to be imposed in order to seamlessly implement BC in the energy market without any confusions.

## Data Availability

The datasets used and/or analysed during the current study are available from the corresponding author upon reasonable request.
